# Solid-Contact Electrode with Composite PVC-Based 3D-Printed Membrane. Optimization of Fabrication and Performance

**DOI:** 10.3390/s21144909

**Published:** 2021-07-19

**Authors:** Bartosz Bartoszewicz, Andrzej Lewenstam, Jan Migdalski

**Affiliations:** Faculty of Materials Science and Ceramics, AGH-University of Science and Technology, Al. Mickiewicza 30, PL-30059 Cracow, Poland; bartoszbartosz92@o2.pl (B.B.); migdal@agh.edu.pl (J.M.)

**Keywords:** reference electrode, solid contact, PVC-based composite, 3D printing, optimized fabrication

## Abstract

Intense interest in reference electrode design and fabrication has recently been enriched with the application of 3D printing of electrodes with salt-loaded PVC membranes. This type of material is attractive in sensor technology and is challenging to implement in 3D. In this report, several improvements and simplifications in the technology were focused on and supported by a fundamental electrochemical characterization.

## 1. Introduction

The invention of solid-contact ion-selective electrodes has opened an avenue covering fabrication, application, and theory in ion-sensor technology [1,2,3,4,5,6]. The reference electrode, a counterpart in electrochemical measurement, was initially treated as an issue of its own. Indeed, the materials used and the fabrication technology, the methods of characterization and testing have been, and are, distinctly different, see: [7]. However, new expectations and a need for ion-monitoring have inspired a boom of interest in designing reference electrodes, which is reflected in numerous reports [8,9,10,11,12,13,14,15,16,17,18,19,20].

Of particular importance is the fabrication method of the solid-contact reference electrodes by injection moulding [21]. For the first time, the application of thermoplastic polymers, such as poly (vinyl acetate) (PVAc) or poly(propylene) (PP), with a high content of equitransferent salts, was demonstrated [13,15]. This method is fast and straightforward, which allows for fabrication in one injection step of the electrodes of different shapes and dimensions. Additionally, the electrochemical reference electrode role can embody several ion-selective electrodes with poly (vinyl chloride) (PVC)-based membranes, forming an integral multiplatform [2]. The success of this approach founded a perspective for the application of 3D printing. With 3D printing, in which the moulding forms are redundant, one could expect a cost reduction. However, the open challenge was whether 3D printing could be applied for a PVC-based electroactive membrane highly loaded with equitransferent salts. PVC is the most frequently used polymer for solid-contact ion-selective plastic membrane sensors. Moreover, PVC can also be used for designing solid contact reference electrodes by drop-casting, as has recently been shown [22,23]. Our group has contributed to meeting the above challenge by reporting on the 3D printing fabrication of a PVC solid-contact reference electrode (SC REF 3D) [24]. This novel development earned recognition as the first-ever true 3D-printed reference electrode [12]. Whatever the method of SC REF fabrication, the primary reason for the response is in boundary potential between the polymer-salt PVC-KCl composite membrane and the sample, which is dominated by hindered salt dissolution [13,21,22,24].

In this report, we focus on the essential constrictions on the way to obtaining better metrological parameters. We provide information on essential improvements to this novel technology. We show how SC REF 3D can be further optimized in respect of the divergence of base-line formal potentials, response stability, and lifetime.

## 2. Materials and Methods

### 2.1. Reagents

Silver 99.9 in the form of a wire (diameter 3 mm) or tape (1 mm thick, 3 mm wide) was obtained from Hopea, Warsaw, Poland. Prior to using the REF 3D electrode, the silver was polished and ultrasonically cleaned in methanol and water. Next, the silver was coated with silver chloride by electro-oxidation in 1 M KCl solution by applying a current density of 3.2 mA cm^−2^ for 10 min.

Acrylonitrile butadiene styrene (ABS) was obtained from Noctuo, Gliwice, Poland.

Silver nanowires of diameter 175 nm × 20–50 nm as 0.5% suspension in isopropanol (Sigma—Aldrich, Steinheim, Germany) were used as received. Tetrahydrofuran (THF) from Sigma-Aldrich was double-distilled before use.

Bis(2-ethylhexyl)-sebacate (DOS) and PVC were obtained from Fluka.

The other p.a. compounds of p.a. purity (KCl, NaCl, NaBr, NaHCO_3_, NaOH, KOH, HCl, CH_3_OH) were obtained from POCh, Poland, or Merck, Germany, and were used as received. 

Water re-distilled from quartz was used to prepare the solutions. All solutions with concentrations lower than 0.01 mol dm^−3^ were prepared just before use.

### 2.2. Preparation of the PVC/KCl Composite for Membrane 3D Printing

The homemade composite used as an active reference membrane was made from 50 weight parts KCl, 32.5 parts PVC, 1.5 parts heat stabilizer, and 16 parts diethylhexyl terephthalate plasticizer [20].

### 2.3. Preparation of the Flat Solid Contact

A flat Ag tape with a thickness of 1 mm and 3 mm was coated with AgCl in preparation for the contact with the PVC/KCl composite. The AgCl layer length was 7 mm, and the total length of the tape was 35 mm.

### 2.4. Preparation of the Reference Cocktails

2.06 g bis(2-ethylhexyl)-sebacate (DOS) and 1.24 g of PVC were dissolved in 25 mL of freshly distilled THF. To prepare the AgCl/KCl based reference cocktail, silver chloride and potassium chloride were first dried, mixed in molar ratio 1:5, and thoroughly ground. Next, 1.5 mL of PVC/DOS solution in THF and 0.1 mL silver nanowires in isopropanol were added to 0.224 g of AgCl/KCl mixture [18,19].

### 2.5. Apparatus

#### 2.5.1. 3D Printer

A custom-made printing device with combined classical fused deposition modelling (FDM) module and a specifically designed module able to produce enough pressure force for composite printing was used to create both the body and the electroactive composite membrane. The FDM module had a typical construction that used a wide range of materials and temperatures commonly used in 3D printing. The special module consists of a stainless-steel piston and extrusion die with a 2 mm diameter neck shaped to build up pressure. The extrusion die can be heated up to 300 °C, and a piston can create pressure of around 200 atmospheres. Both modules share a common Z axis but separate the X and Y-axis. Thanks to this approach, both modules can be used in a single process of printing for two materials, one after the other.

The printer is controlled by a Duet Wifi 2 master board and can be operated with a touch-screen panel, produced by Duet3D Limited, Great Britain, or remotely from a PC. It is programmed using G-code standard and procedural scripts.

#### 2.5.2. Electrochemical Measurements

The potentiometric measurements were performed using a homemade 16-channel set-up. The input impedance was greater than 10^13^ Ohms, and the input current was lower than 20 fA for each of the 16 inputs and the reference electrode input. The multi-channel potential-meter was coupled with a personal computer equipped with a 16-bit resolution data acquisition card PCI DAS 6014 (Computer Boards; Norton, MA, USA) or a 16-bit resolution data acquisition card USB1606G (MC Measurements Computing, Norton, MA, USA) and custom-made software. In typical conditions, the potential resolution was better than 0.02 mV.

Electrochemical Impedance Spectra (EIS) were usually recorded in 0.1 M KCl solutions using an Autolab PGSTAT302N analyzer equipped with a Frequency Response Module FRA32M. Impedance spectra were recorded in the frequency range of 500 kHz to 0.01 Hz under open-circuit potential with 20 mV sinusoidal excitation signals. All experiments were performed at room temperature (22–24 °C).

The single-junction (REF201 Metrohm; Herisau, Switzerland) was used as the reference electrode in the potentiometric and EIS measurements. The Pt wire or GC rod was used as an auxiliary electrode in the EIS measurements. All experiments were performed at room temperature (22–24 °C).

#### 2.5.3. SEM Images

The composite morphology was studied with a NOVA NANO SEM 200 Ultra Scanning Electron Microscope (FEI EUROPE COMPANY, Hillsboro, OR, USA), in conjunction with an EDS analyzer SiLi (EDAX Inc., Mahwah, NJ, USA) equipped with Genesis Spectrum Version 6.531. Two composite membranes made of the native PVC loaded with KCl filament before 3D printing and after 3D printing at 225 °C were investigated. Both membranes were conditioned in 0.1 KCl and dried before SEM imaging. The composite samples were cooled in liquid nitrogen and then broken for SEM imaging. Before SEM image registration, the surface of the composite was covered with carbon.

## 3. Results and Discussion

### 3.1. Sketching the Problems

**Figure 1:**
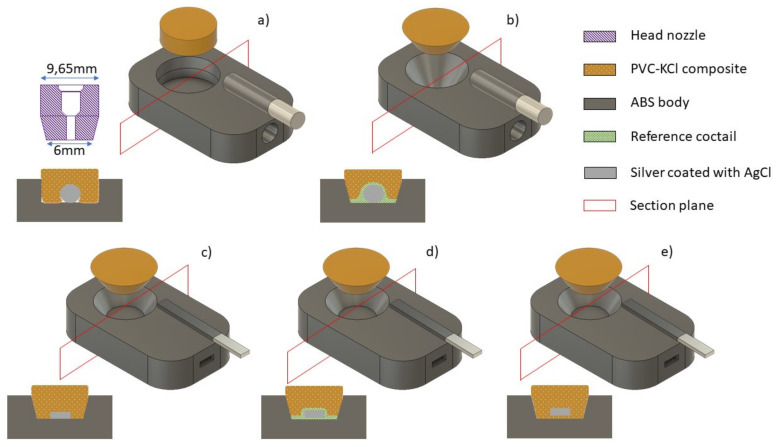
Sources in 3D fabrication deficiencies and their removal: (**a**,**c**)—the Ag/AgCl rod (**a**) or tape (**c**) was inserted into the pre-printed hole, and the cylindrical (**a**) or conical (**c**) crater was filled with PVC-KCl composite. (**b**,**d**)—some of the reference cocktail was placed in the crater, and the Ag/AgCl rod (**b**) or tape (**d**) was inserted into the pre-printed hole. After solvent evaporation, the crater was filled with PVC-KCl composite (**e**)—the conical crater was filled with PVC-KCl composite. The Ag/AgCl tape was immediately inserted into the hot unhardened plastic composite. In the upper left corner: the lower part of the nozzle (neck diameter 2 mm). Crosscuts made by selection planes visualize the shape of the crater, solid contact, and its position in the composite.

Without a doubt, the fabrication of all-solid-contact electrodes by 3D printing is a novelty in the area of electrochemical sensors, as noted by some authors [1,12]. The first report focused on the general description of the fabrication. In this article, we would like to address some operational problems influencing analytical performance, which could be randomly observed and systematically circumvented by redesigning the SC REF, as illustrated in Figure 1.

### 3.2. Increasing the Adhesion of the Composite to the Embodying Neck

The effect of adhesion was addressed by changing the geometry of the crater from a cylindrical (Figure 1a) to a conical shape (Figure 1b–e) and by optimizing the temperature of the printer head nozzle. 

A truncated cone crater was dimensioned to match the geometry of the tip of the composite printing nozzle. The reason for this change was anticipated improvement of the printing quality, reducing the possibility of empty space formed between the composite and ABS body, as well as between the composite and Ag/AgCl rod or tape solid contact. Enhanced adhesion of the composite to ABS and Ag/AgCl was expected.

The effects of temperature and its influence on the composite were studied in the range of 200–240 °C. The nozzle temperature was selected to avoid the risk of PVC decomposition and slow down the composite printout for better coverage. The temperature of 225° C was selected as the optimal for printing. Below 200 °C, the filament is too viscous to achieve printout, and above 240 °C, the irreversible decomposition of PVC occurs.

**Figure 2:**
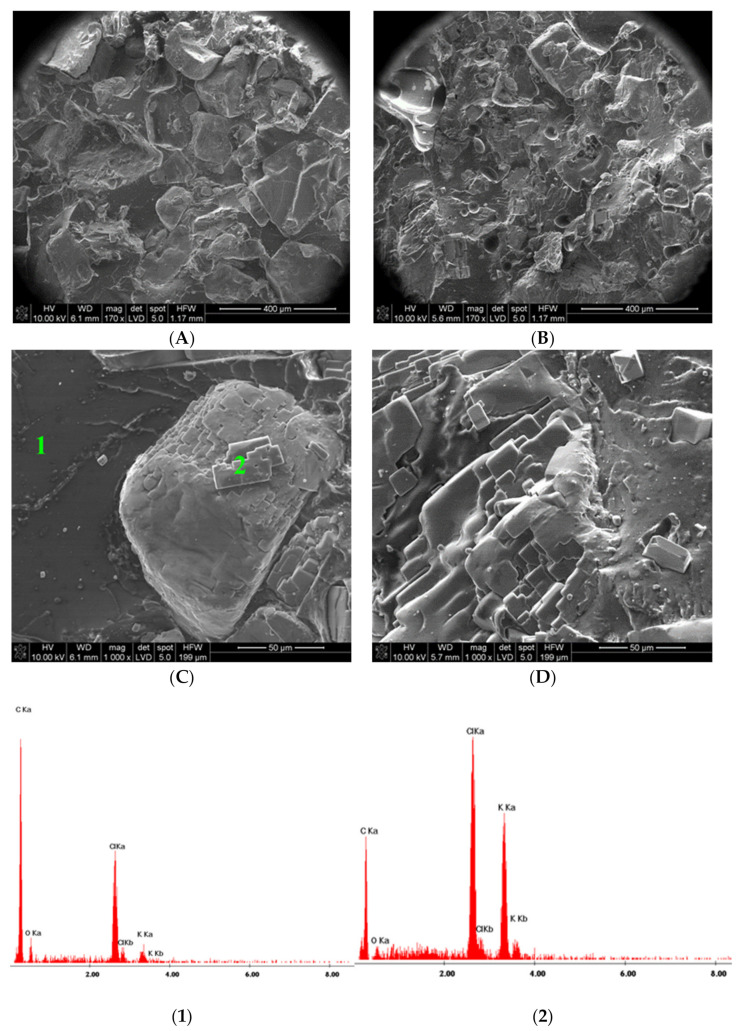
Scanning electron micrograph of the composite membrane conditioned in 0.1 KCl. Images taken with two different resolutions of 400 µm/div (**A**,**B**) and 50 µm/div (**C**,**D**), for the native filament before 3D printing (**A**,**C**) and the filament after 3D printing (**B**,**D**). EDAX spectra taken for points **1** and **2** as indicated in the photo “**C**”. (**1**)—point **1** PVC matrix covered with KCl dust, (**2**) point **2** —KCl crystal.

The SEM images of the composite structure obtained directly from the native filament to that fabricated by 3D printing indicate that the porous structure responsible for electrochemical responses is unchanged (Figure 2). However, the dimensions of the KCl crystals were decreased, and distribution in the membrane was enhanced.

### 3.3. Increasing Adhesion of the Composite to Solid Contact

Initially, after printing the ABS body, an Ag/AgCl contact in a rod shape was inserted into the pre-printed hole, and the cylindrical crater was filled with PVC/KCl composite. 

Using this method, a chance for unfavourable “empty” space was created, symptomatic of an increase in ohmic resistance and solution-to-solution memory effects.

Some of the electrodes prepared in this way exhibited deteriorated functioning after a long period of use, obviously due to the voids in the SC-composite interface filled with the residual sample solution and uncontrolled memory effects. The adhesion of the composite to the internal contact directly influences the charge transfer at this interface and its accompanying resistance.

In order to increase the contact surface, the originally used Ag/AgCl wire (Figure 1a,b) was replaced with a flat Ag/AgCl tape with a thickness of 1 mm and a width of 3 mm (Figure 1c–e). The strip length was approximately 35 mm, and a section of Ag coated with AgCl was in contact with the PVC/KCl composite over a length of approximately 7 mm.

Initially, the original procedure was employed for electrode fabrication, i.e., after printing the ABS body, an Ag/AgCl tape was inserted, and the conical crater was filled with PVC/KCl composite (Figure 1c). This way, the possibility of the formation of “empty space” was reduced, but the bottom part of the Ag/AgCl tape did not have contact with the composite.

To eliminate this problem, new procedures were employed. In the first, some of the THF/DOS/PVC/KCl/AgCl reference cocktail [18,19] in the form of suspension was placed into a conical crater, and after that, the Ag/AgCl wire or tape was inserted. This way, both sides of the Ag/AgCl wire/tape were covered with a reference cocktail. After solvent evaporation (about 24 h), the crater was filled with PVC/KCl composite. It was expected that the use of the DOS/PVC/KCl/AgCl cocktail ensures good electrical contact, which was proven experimentally.

**Figure 3:**
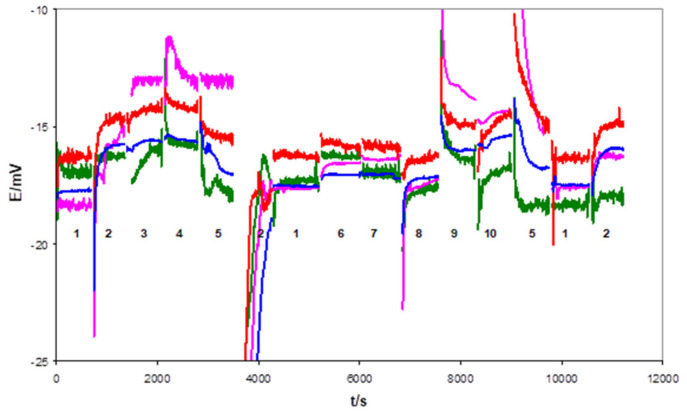
Potential changes of the 1c type electrodes recorded during multi-solution testing subsequently performed in indicated solution: 1—3 M KCl, 2—H_2_O, 3—0.01 M NaCl, 4—0.01 M KCl, 5—0.01 M HCl, 6—0.1 M NaCl, 7—0.1 M KCl, 8—0.1 M NaBr, 9—0.1 M NaHCO_3_, 10—0.001 M KOH. The mean potential values for these electrodes were equal to: −15.95 ± 1.04 mV, −15.62 ± 1.85 mV, −15.46 ± 1.06 mV, and −16.75 ± 1.02 mV.

**Figure 4:**
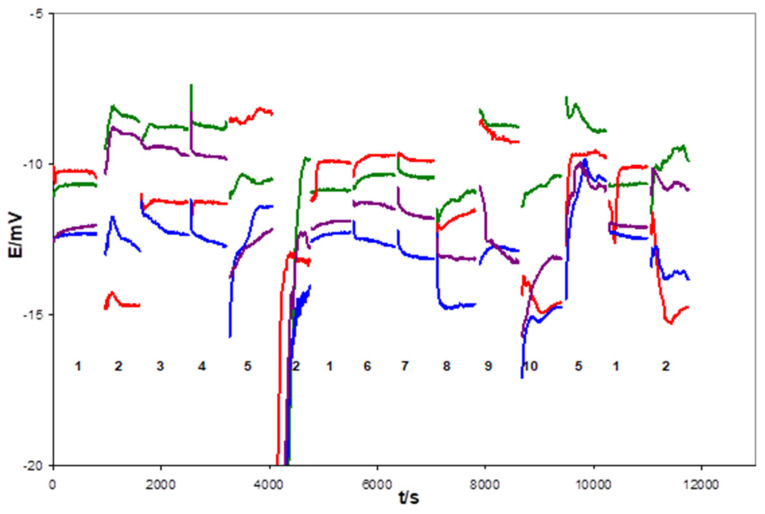
Potential changes of the type electrodes recorded during multi-solution testing subsequently performed in indicated solution: 1—3 M KCl, 2—H_2_O, 3—0.01 M NaCl, 4—0.01 M KCl, 5—0.01 M HCl, 6—0.1 M NaCl, 7—0.1 M KCl, 8—0.1 M NaBr, 9—0.1 M NaHCO_3_, 10—0.001 M KOH. The mean potential values for these electrodes were equal to: −9.91 ± 0.88 mV, −11.30 ± 2.16 mV, −12.88 ± 1.12 mV, and −11.61 ± 1.30 mV.

After 1 week of conditioning in 0.1 M KCl, the electrodes with flat internal contact, uncovered (1c) and covered (1d) by the mediating layer, functioned properly—which was proven by multi-solution testing protocol (MSP) [13]. For example, Figure 3 and Figure 4 show the potential changes recorded for 1c type and 1d type electrodes in the sequence of the selected solution. However, significant differences in the resistance of the electrodes were observed between these two groups, as confirmed by the EIS measurements. Usually, resistances measured for electrodes from the 1d group did not exceed a few dozen kOhms, whereas the 1c group was in the range of a few MOhms. For this reason, more noisy potential signals were recorded during MSP of 1c type electrodes, as can be seen from a comparison of Figure 3 and Figure 4.

### 3.4. Printing of Optimized SC REF 3D

The use of the DOS/PVC/KCl/AgCl cocktail ensures good electrical contact, makes the electrodes’ preparation relatively laborious and requires several consecutive operations at intervals of several hours.

**Figure 5:**
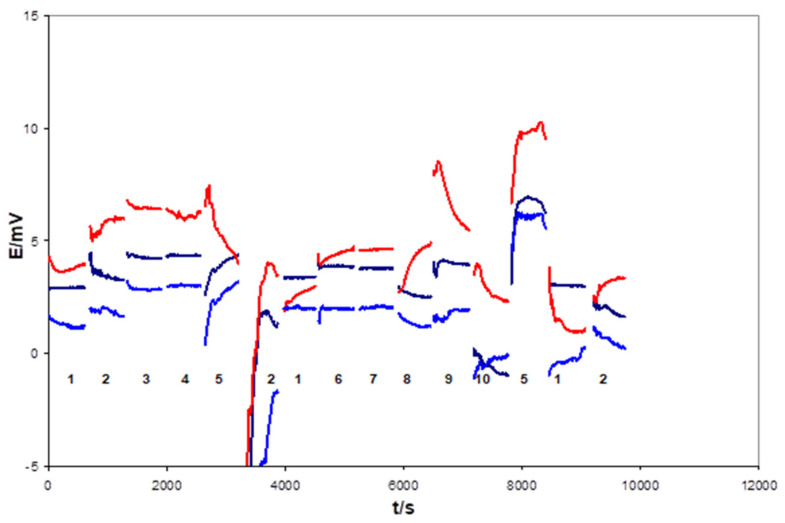
Potential changes of the 1e type electrodes recorded during multi-solution testing subsequently in indicated solution: 1—3 M KCl, 2—H_2_O, 3—0.01 M NaCl, 4—0.01 M KCl, 5—0.01 M HCl, 6—0.1 M NaCl, 7—0.1 M KCl, 8—0.1 M NaBr, 9—0.1 M NaHCO_3_, 10—0.001 M KOH. The mean potential values for these electrodes were equal to 3.17 ± 1.65 mV, 4.59 ± 2.03 mV, and 1.67 ± 1.69 mV.

Therefore, attempts were made to reduce the electrode preparation time while ensuring the two-sided contact of the composite with the Ag/AgCl tape. The conical crater in the ABS body was filled with the composite, and immediately the Ag/AgCl tape was inserted (pressed) into the hot, unhardened plastic composite so that the composite sticks to the lower and upper surfaces of the Ag/AgCl tapes. Such a method was found to offer the fastest and easiest way to manufacture REF 3D electrodes. The analytical results showed that the electrodes obtained in this way function properly, although they require a longer conditioning period (compared to electrodes with a DOS/PVC/KCl/AgCl cocktail). After 3 weeks of conditioning, the electrodes made in this way (1e type electrodes) worked properly, and their resistance was close to that of the electrodes using a cocktail (1d type electrodes). The potential changes of these electrodes recorded in the multi-solution calibrations are shown in Figure 5.

EIS measurements confirmed similar resistances of the 1d and 1e type electrodes. 

**Figure 6:**
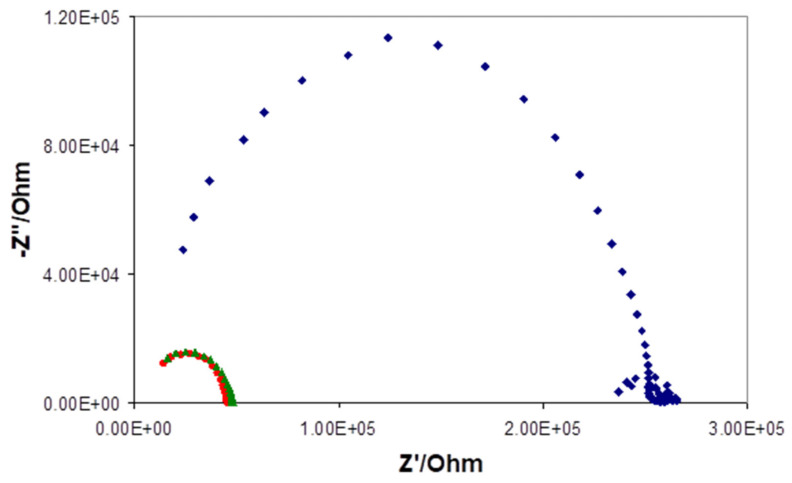

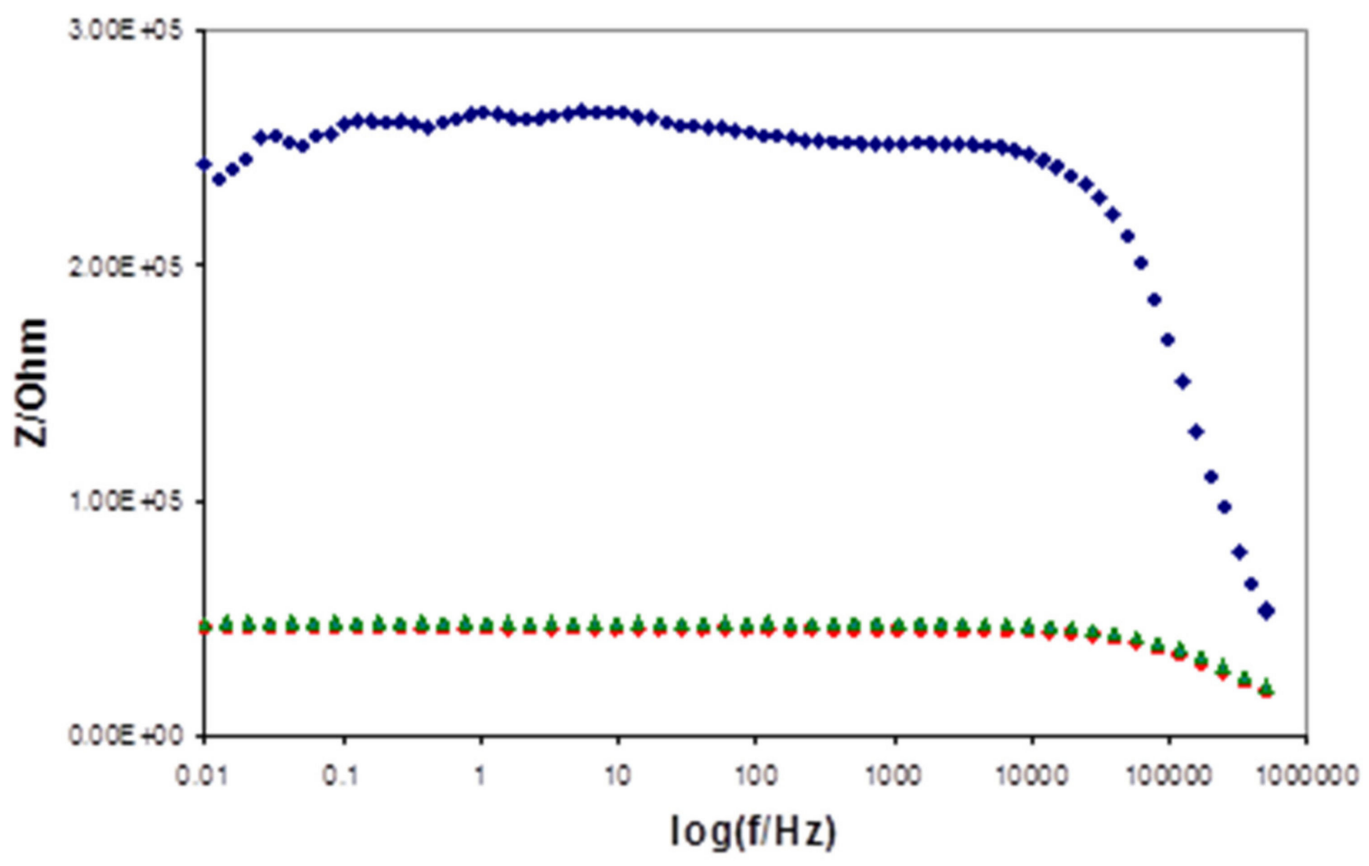
EIS spectra of the 1a type (blue rectangle), 1d type (red circle), and 1e type (green triangle) electrodes recorded in 0.1 M KCl solution under open circuit potential. Frequency range 500 kHz—0.01 Hz, amplitude 20 mV.

For comparison, the EIS spectra recorded for the 1d and 1e type electrodes and the best electrode of older 1a type are shown in Figure 6. The significant decrease in ohmic resistance proves a crucial role of the solid contact-composite interface in the mechanism and quality of an SC REF 3D response.

The group 1d and 1e electrodes also showed very good potential stability during long-term contact with water. For example, the mean value of the potential measured during 45 h of contact with distilled water was equal to (mean ± SD): −8.44 ± 0.28 mV and −7.51 ± 0.56 mV for two electrodes from group 1d. The two electrodes from group 1e were −9.99 ± 0.21 mV and −7.51 ± 0.24 mV, respectively.

These electrodes also exhibited stable potential during continuous 62-day contact with 0.1 M KCl solution. The results of the measurements are collected in Table 1.

**Figure 7:**
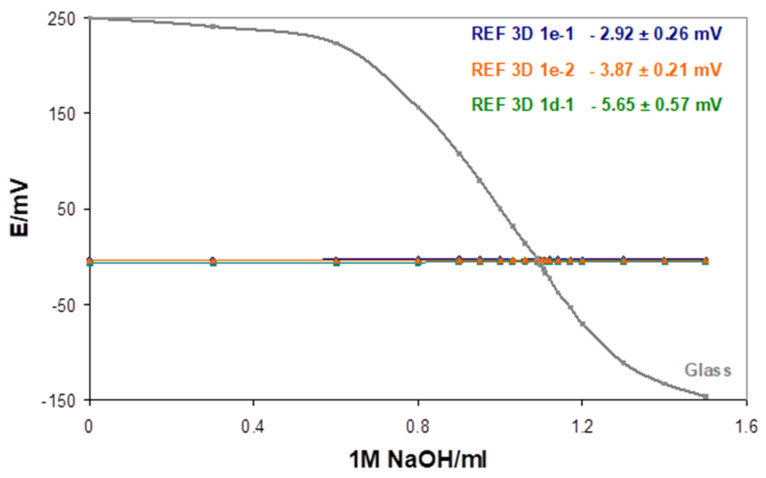
Potential changes of the glass electrode and three REF 3D electrodes (two 1e types and one 1d type) recorded during titration 50 mL 0.02 M HCl with 1 M NaOH.

For analytically relevant information, potential changes of SC REF 3D were recorded in titration, 50 mL 0.02 M HCl with 1 M NaOH (see Figure 7). The mean potential values during titration were: −2.92 ± 0.26 mV and −3.87 ± 0.21 mV for two electrodes of 1e type and 5.65 ± 0.57 mV for 1d type electrode.

## 4. Conclusions

The obtained results showed that the SC REF-3D electrodes with a modified crater shape and the use of the DOS/PVC/KCl/AgCl cocktail to improve the electrical contact (Figure 1b,d) are more reliable and work better than those in which the contact Ag/AgCl wire was inserted into the cylindrical crater. The use of a conical crater and solid contact in the form of Ag/AgCl strip tape inserted into the hot, unhardened plastic composite resulted in the fast fabrication of an SC REF 3D with excellent metrological properties.

## Figures and Tables

**Table 1:** Potential of the electrodes 1d and 1e recorded during continuous contact with 0.1 M KCl solution.

Days of Soaking	Electrode 1e-1 (mV)	Electrode 1e-2 (mV)	Electrode 1d-1 (mV)	Electrode 1d-2 (mV)	Electrode 1d-3 (mV)
1	−2.4	1.7	−1.4	−1	0.4
5	−2.5	1.7	−1.6	−1.7	0.1
9	−2.8	1.7	−1.6	−1.7	0.1
10	−2.4	1.7	−1.8	−2.1	−0.1
12	−2.5	2.6	−1.5	−1.8	−0.1
13	−2.9	1.6	−1.6	−1.9	−0.1
16	−2.9	1.8	−1.6	−1.4	−0.2
17	−2.2	2.9	−1.8	−1.7	0.0
19	−2.3	2.4	−1.7	−1.5	−0.1
22	−2.3	2.0	−1.9	−1.3	0.1
23	−2.6	2.0	−2.2	−2.3	−0.2
30	−2.9	1.5	−2.7	−2.6	−1.0
38	−3.2	1.0	−3.1	−2.9	−1.1
47	−3.6	1.3	−3.3	−3.3	−0.9
49	−3.0	1.7	−2.8	−3.1	−1.0
62	−2.9	0.9	−2.9	−3.4	−3.2
**mean**	−2.71	1.78	−2.09	−2.11	−0.46
**SD**	0.38	0.53	0.64	0.75	0.87
